# Supplementary figures and images for: Characterizing COVID-19 clinical phenotypes and associated comorbidities and complication profiles
Source: PLoS One. 2021 Mar 31;16(3):e0248956. doi: 10.1371/journal.pone.0248956 (PMC8011766; doi:10.1371/journal.pone.0248956)

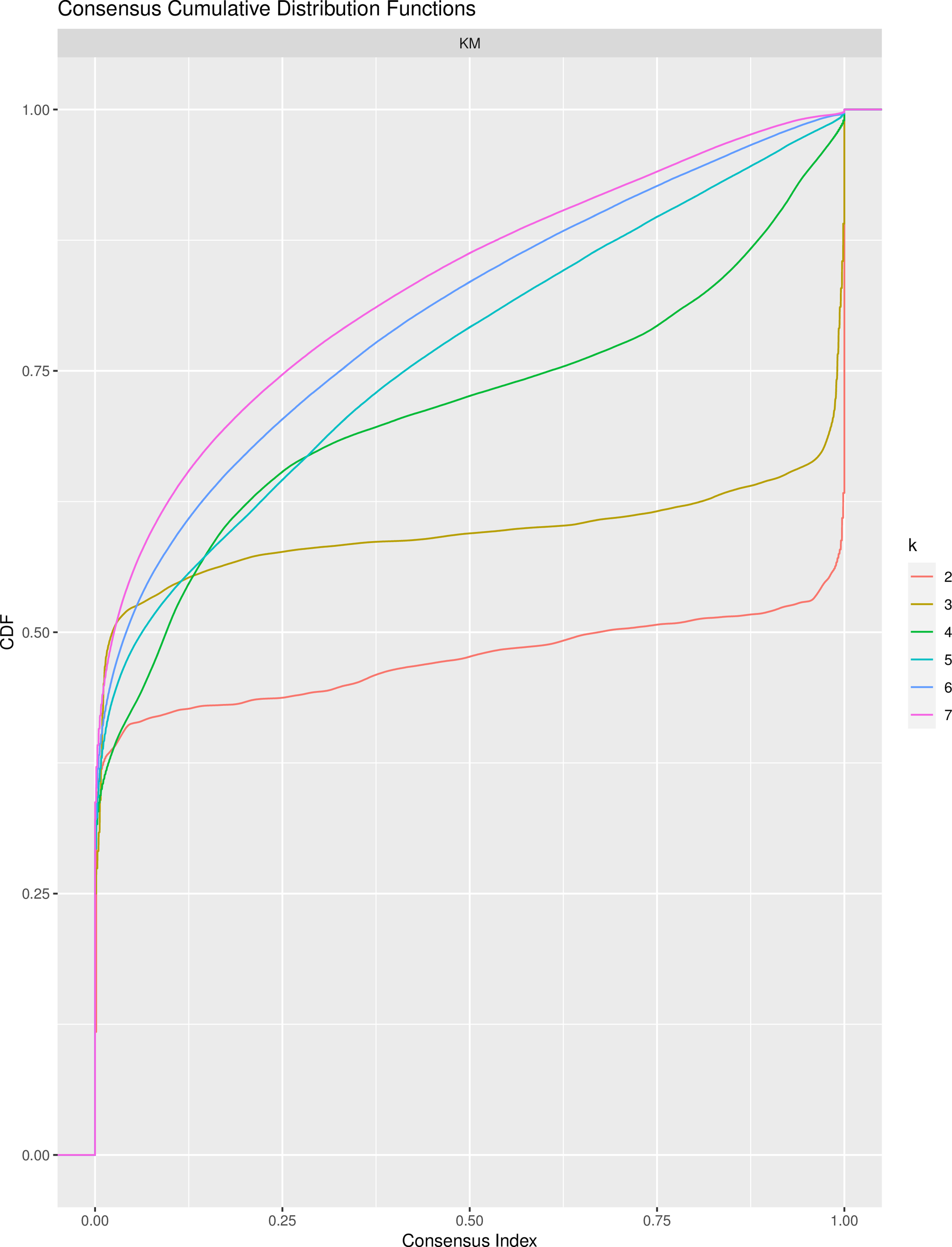

Supplement: S1 Fig — Cumulative distribution functions (CDF) for a randomly selected imputed dataset are shown. A range of phenotypes (2–7) were considered, and the optimal choice of phenotypes is 3. (TIF) [file pone.0248956.s001.tif]

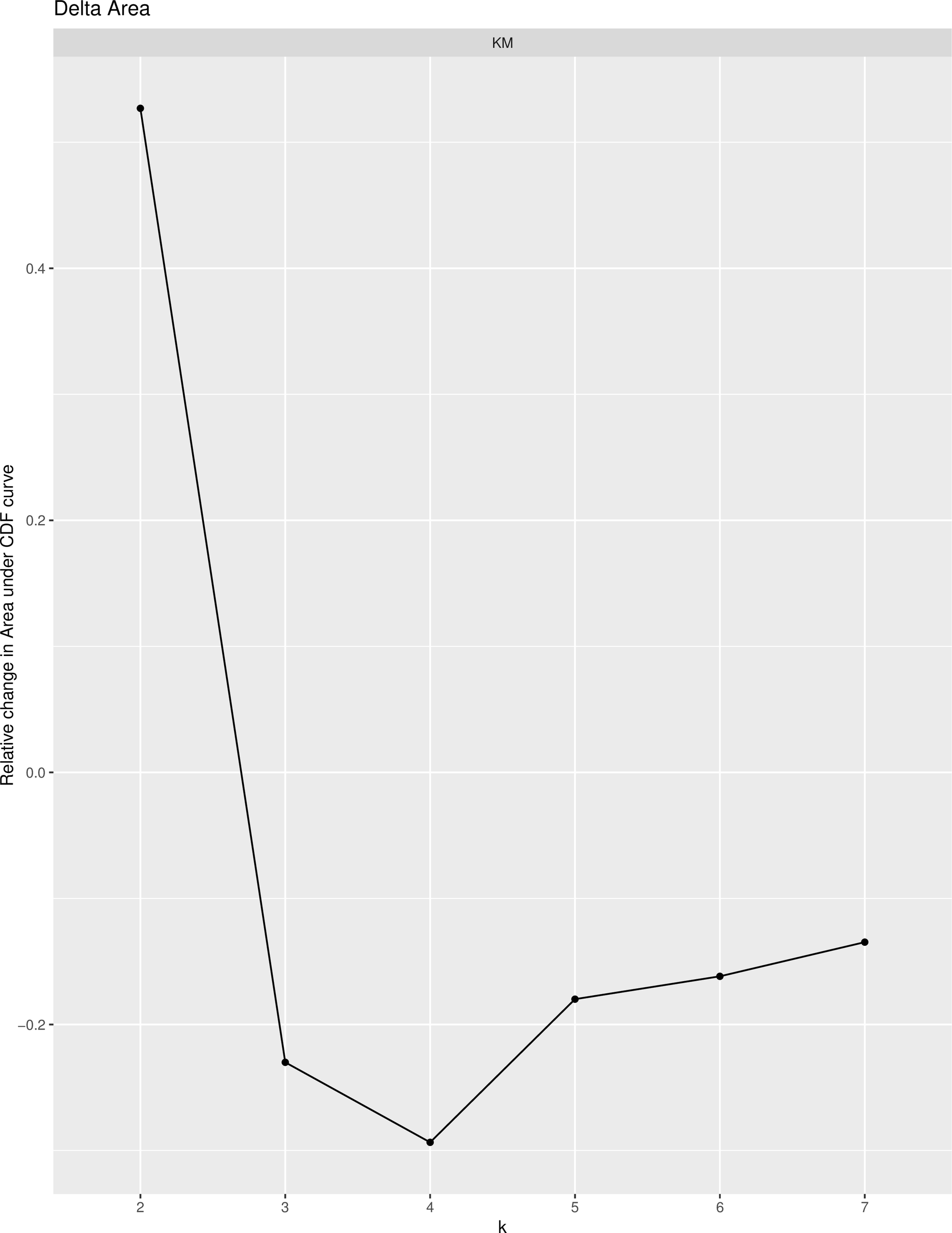

Supplement: S2 Fig — The relative change in delta area under the cumulative distribution function is shown for the range of phenotypes (k = 2–7) for a randomly selected imputed dataset. The optimal choice of phenotypes is 3. Abbreviations: CDF (cumulative distribution function). (TIF) [file pone.0248956.s002.tif]

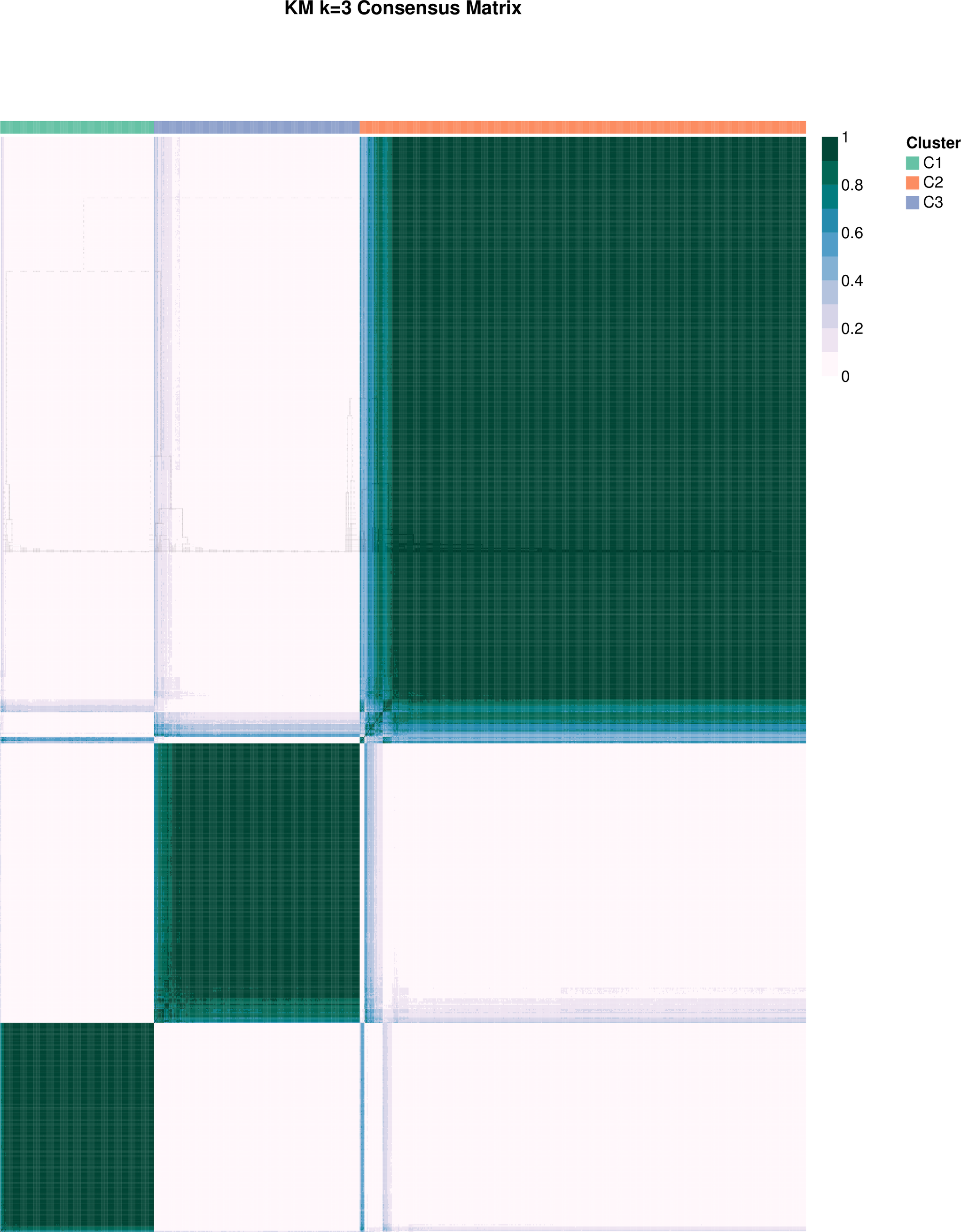

Supplement: S3 Fig — A consensus matrix heatmap is shown for a randomly selected imputed dataset clustered into 3 phenotypes. The heatmap allows visualization of consensus cluster assignments to evaluate cluster stability. Darker shades of green indicate higher stability. (TIF) [file pone.0248956.s003.tif]

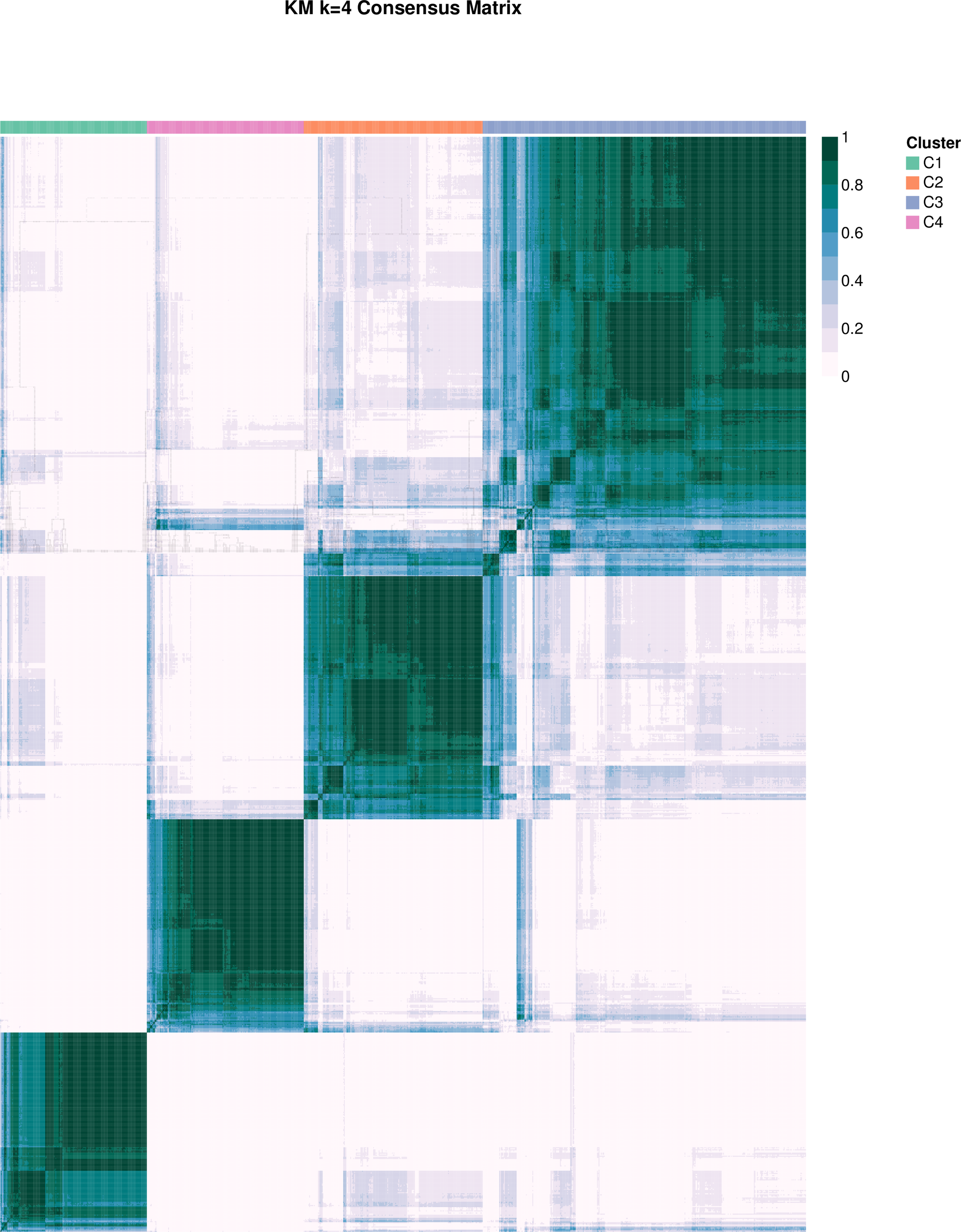

Supplement: S4 Fig — A consensus matrix heatmap is shown for a randomly selected imputed dataset clustered into 4 phenotypes. The heatmap allows visualization of consensus cluster assignments to evaluate cluster stability. Darker shades of green indicate higher stability. The choice of 4 clusters shows less stability than 3 clusters (see S3 Fig). (TIF) [file pone.0248956.s004.tif]

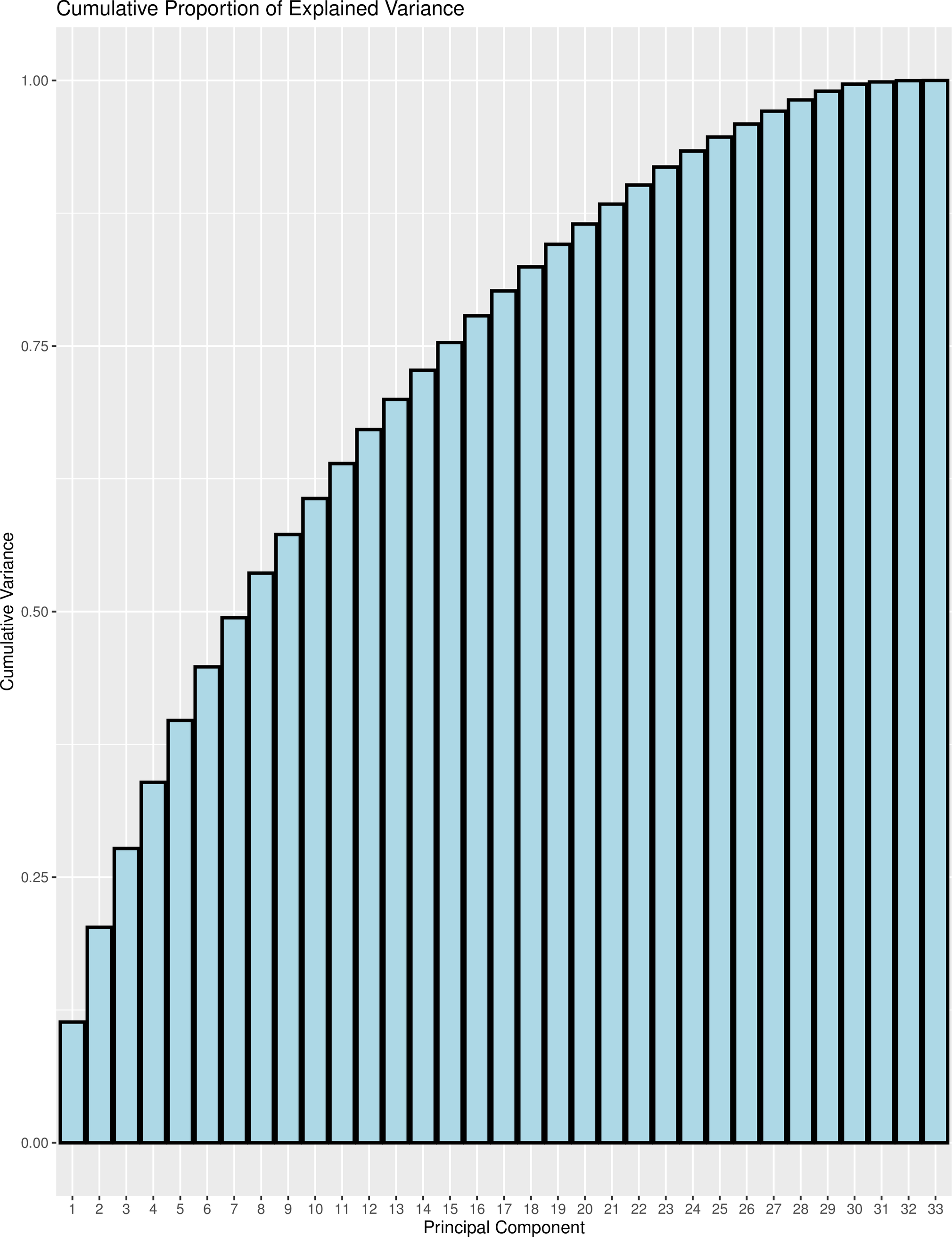

Supplement: S5 Fig — The proportion of variance explained by each principal component is summed over all principal components. For example, PC1 and PC2 cumulatively explain 20% of the variation in the dataset. Abbreviations: PC1 (principal component 1); PC2 (principal component 2). (TIF) [file pone.0248956.s005.tif]

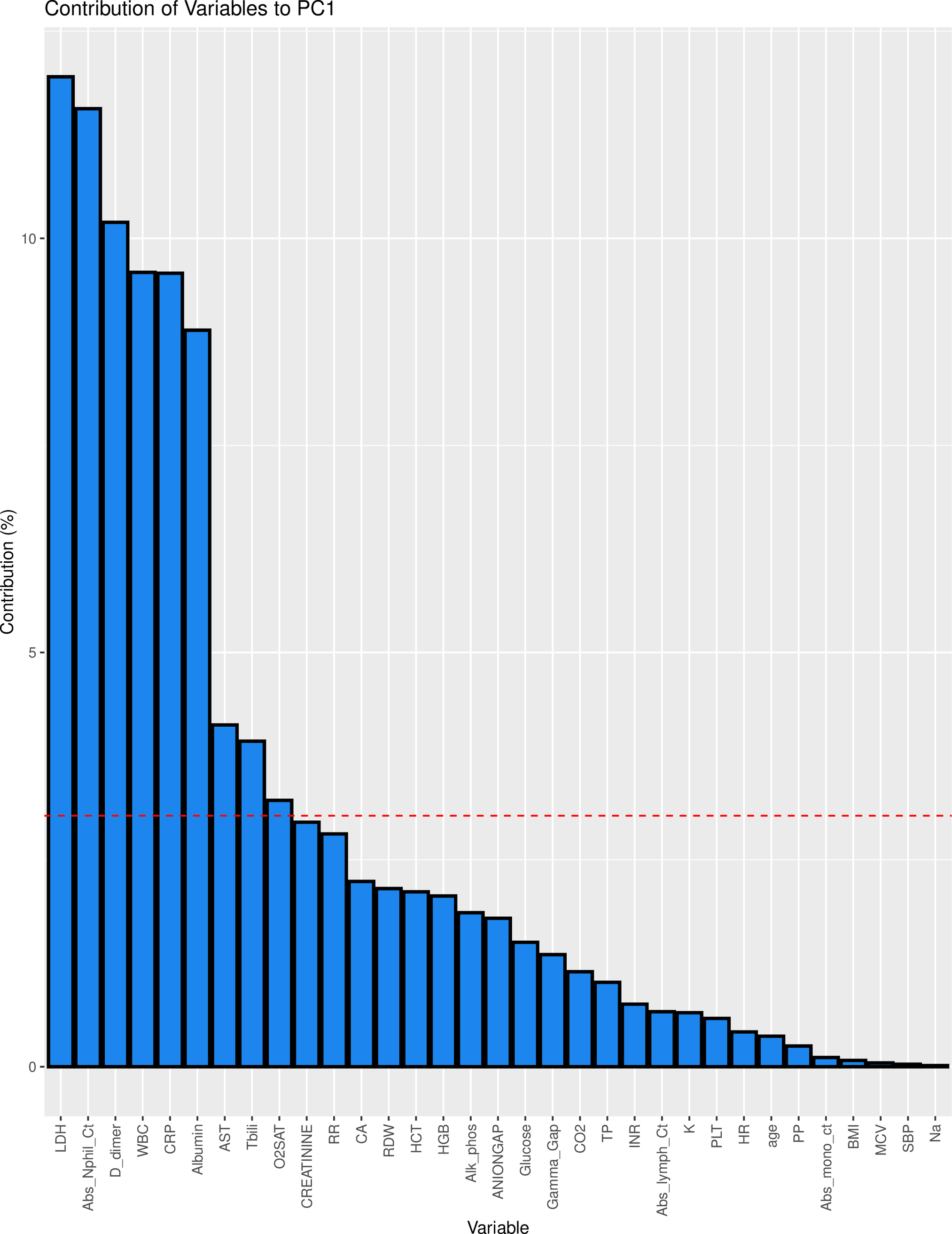

Supplement: S6 Fig — The contributions of each of the 33 variables used in the clustering to principal component 1 are shown. The red line marks the expected average contribution of each variable if the contributions of the variables were uniform across the dataset. Variables contributing most to the observed pattern in PC1 are D-dimer and albumin. Abbreviations: PC1 (principal component 1); Abs_Nphil_Ct (absolute neutrophil count); LDH (lactate dehydrogenase); CRP (C-reactive protein); WBC (white blood cell count); HCT (hematocrit); HGB (hemoglobin); Tbili (total bilirubin); RDW (red cell distribution width); AST (aspartate aminotransferase); Alk_phos (alkaline phosphatase); RR (respiratory rate); CA (calcium); TP (total protein); INR (internal normalized ratio of prothrombin time); CO2 (carbon dioxide); K (potassium); O2SAT (oxygen saturation); BMI (body mass index); PLT (platelet); PP (pulse pressure); Na (sodium); SBP (systolic blood pressure); Abs_mono_ct (absolute monocyte count); MCV (mean corpuscular volume). (TIF) [file pone.0248956.s006.tif]

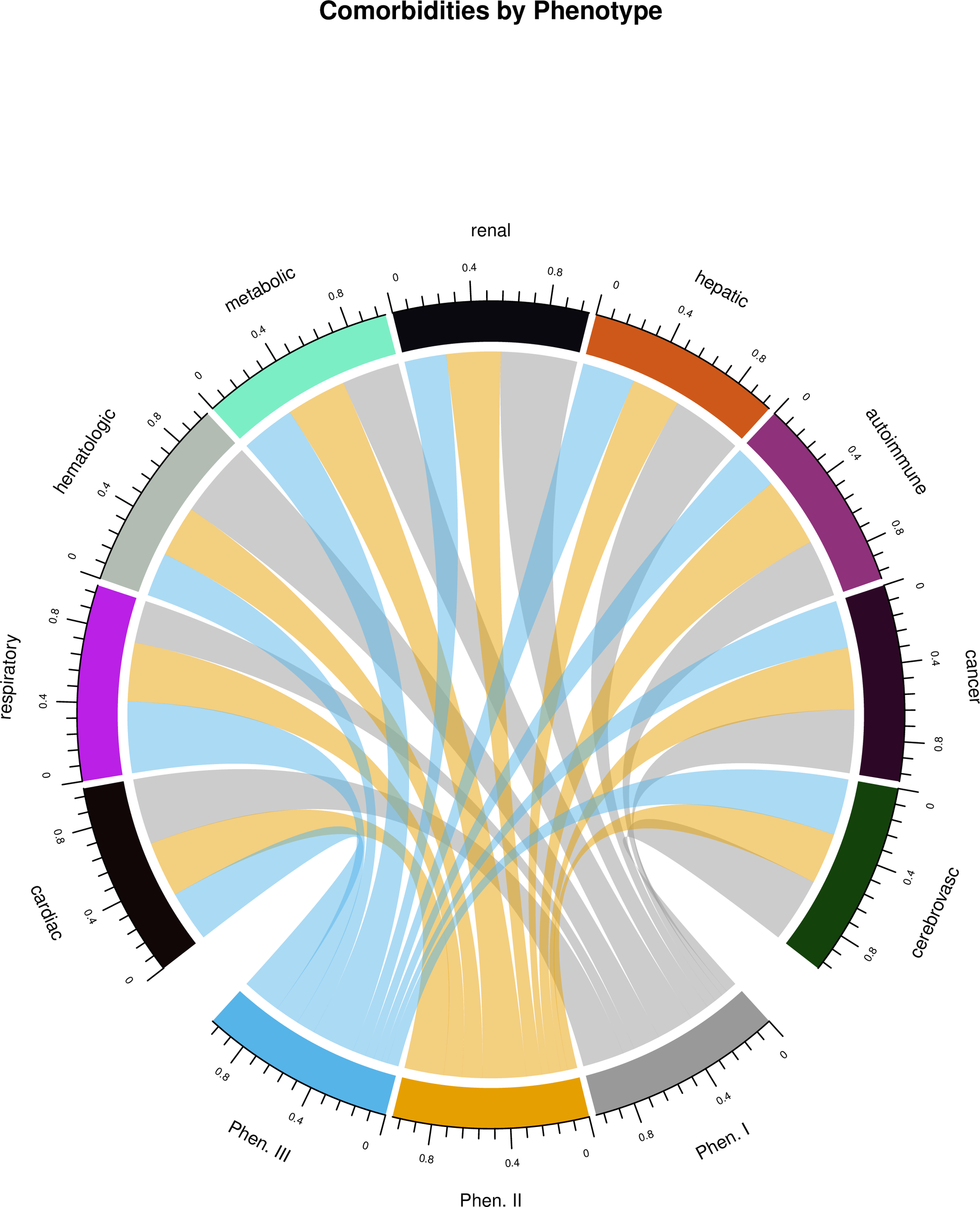

Supplement: S7 Fig — Chord diagram illustrates the prevalence of comorbidities (% observed) for the three clinical phenotypes. (TIF) [file pone.0248956.s007.tif]

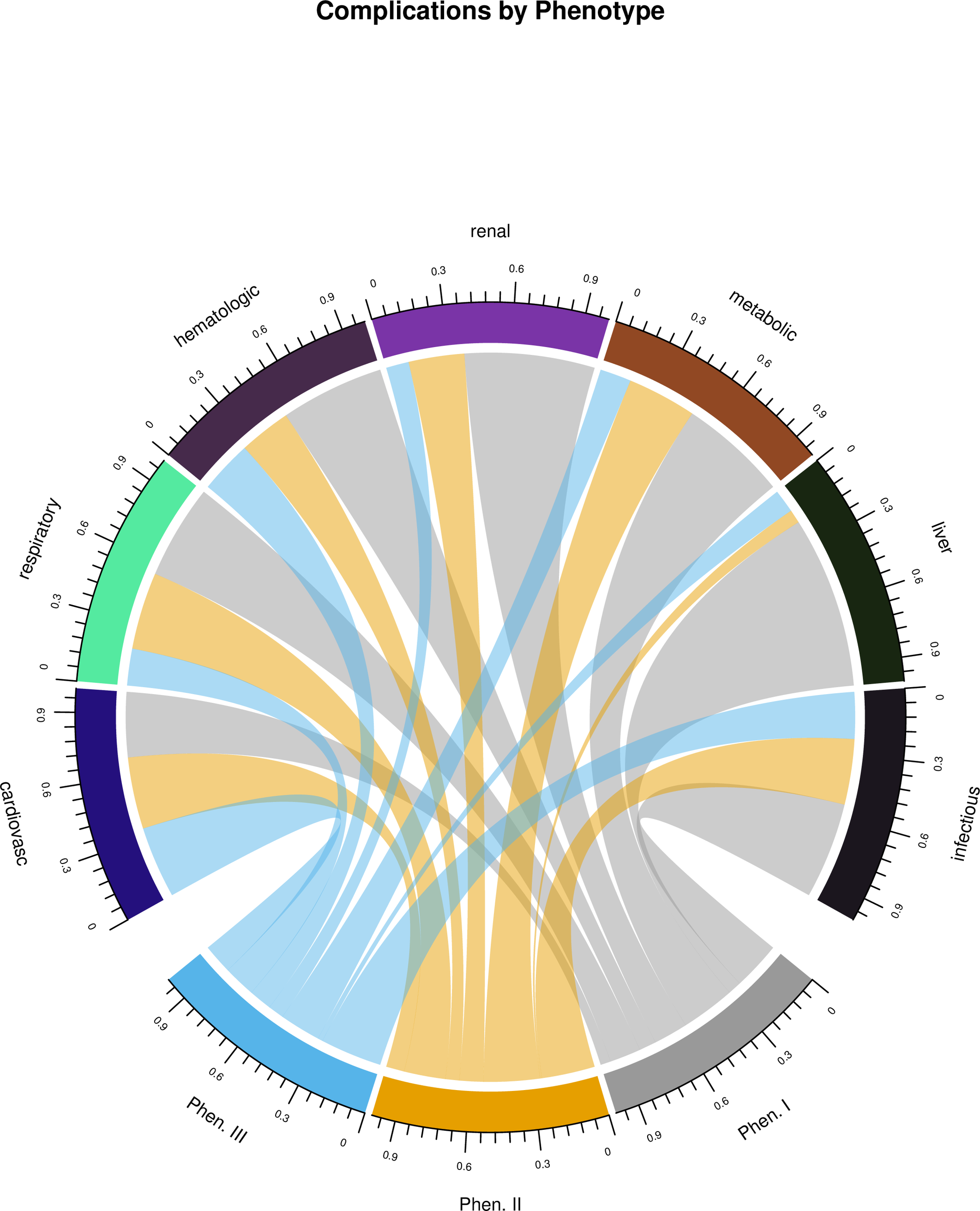

Supplement: S8 Fig — Chord diagram illustrates the prevalence of complications (% observed) for the three clinical phenotypes. (TIF) [file pone.0248956.s008.tif]

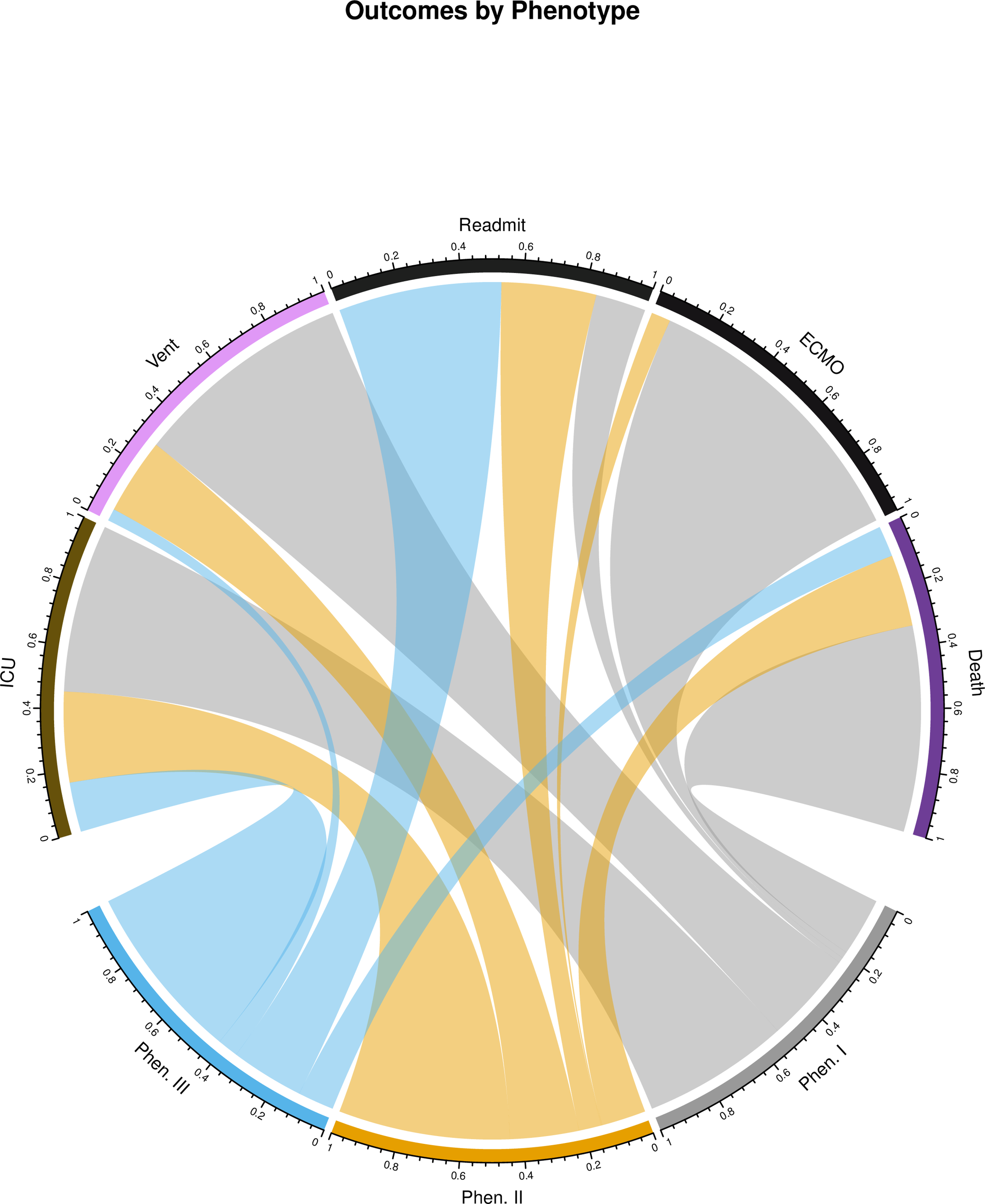

Supplement: S9 Fig — Chord diagram illustrates the prevalence of clinical outcomes (% observed) for the three clinical phenotypes. Abbreviations: ICU (intensive care unit); Vent (mechanical ventilation); Readmit (readmission to hospital or ICU); ECMO (extracorporeal membrane oxygenation). (TIF) [file pone.0248956.s009.tif]
