# Supplementary material for: Characterizing COVID-19 clinical phenotypes and associated comorbidities and complication profiles
Source: PLoS One. 2021 Mar 31;16(3):e0248956. doi: 10.1371/journal.pone.0248956 (PMC8011766; doi:10.1371/journal.pone.0248956)
Supplement: S1 Table — (PDF) [file pone.0248956.s010.pdf]

S1 Table. Categories of Comorbidities and ICD 10 Codes used

Category	Diagnosis
**Cardiac**	Hypertension
	Coronary Artery Disease
	Heart Failure with preserved ejection fraction
	Systolic heart failure
	Acute myocardial infarction
	Unspecified heart failure
	Heart Valve
	Pacemaker
	Automatic Implantable Cardioverter Defibrillator
	Ventricular assist device
	Pulmonary hypertension
	Heart Transplant
	Atrial fibrillation / Atrial Flutter
	Ventricular tachycardia / Ventricular fibrillation
	Supraventricular tachycardia
	Cardiac arrest
**Respiratory**	Active Smoker
	Chronic obstructive lung disease
	Interstitial lung disease
	Asthma
	Bronchiectasis
	Lung transplant
**Hematological**	Venous thromboembolism
	Hypercoagulable state
	Lupus Anticoagulant
	Heparin induced thrombocytopenia
	Disseminated intravascular coagulopathy
	Hypocoagulable state
	Idiopathic thrombocytopenia
	Hemophagocytic lymphohistiocytosis
	Sickle Cell disorder
	Thalassemia
**Oncologic**	Any Cancer ICD10 code
**Metabolic/Endocrine**	Non-alcoholic fatty liver disease
	Non-alcoholic steatohepatitis
	Obesity
	Overweight
	Metabolic Syndrome
	Osteoporosis
	Sleep Apnea
	Prior Bariatric Surgery
	Type 2 diabetes mellitus

**Renal**	Chronic kidney disease (any stage)
	End stage renal disease
	Kidney transplant
**Hepatic**	Liver transplant
	Any Liver Disease
**Autoimmune**	Type 1 diabetes mellitus
	Neutropenia
	Any Autoimmune disease
	Inflammatory bowel disease
	Lupus
	Rheumatoid Arthritis

**Comorbidity**	**ICD10 Codes**
Hypertension	R03.0, I10, I16.0, I11.0, I15.2, I12.9, I11.0
T1DM	E10.21, Z96.41, E10.8, E10.9, E10.10, E10.65, E10.9, E10.641, E10.40, E10.22, E10.69, E10.649, E10.*
T2DM	E11.*
Tobacco	Z71.6, Z72.0
CAD	I25.*
HFpEF	I50.30, I50.31, I50.32, I50.33
Systolic HF	I50.20, I50.21, I50.22, I50.23, I50.40, I50.41, I50.42, I50.43
Any Liver Disease	K70.*, K71.*, K72.*, K73.*, K74.*, K75.*, K76.*, K77.*
NAFLD	K76.0
NASH	K75.81, K75.8
Class 2 Obese	Z68.35, Z68.36, Z68.37, Z68.38, Z68.39
Any Obese	E66
Overweight	E66.3, Z68.25, Z68.26, Z68.27, Z68.28, Z68.29
Class 1 Obese	Z68.30, Z68.31, Z68.32, Z68.33, Z68.34
Class 3 Obese	E66.01*, E66.2*, Z68.4*"
History of VTE	I27.82, Z86.711, Z86.718, I82*
Neutropenia	D70*
Hypercoagulable state	D68.5*, D68.6*
HIT	D75.82
DIC	D65*
Hypocoagulable state	D68.0*, D68.1*, D68.2*, D68.3*, D68.4*, D68.8*, D68.9*, D69.0*, D69.1*, D69.2*, D69.4*, D69.5*, D69.6*, D69.8*, D69.9*, D66*, D67*
ITP	D69.3*
HLH	D76
Methemoglobinemia	D74*
Any Autoimmune Disease	M30*, D86*, D89*, I73.00, I73.01, K75.4, M32*, M34.9, M34.8, M35*, M06*, M45.9, M35.3, K51*

Acute MI	I21.*
Any Heart Failure	I50*
COPD	J44*, J41*, J43*
ILD	J84*
Any asthma	J45
Mild intermittent asthma	J45.2*
Mild persistent asthma	J45.3*
Moderate persistent asthma	J45.4*
Severe asthma	J45.5*
Bronchiectasis	J47*
Sickle cell	D57*
Thalassemia	D56*
Prosthetic Heart Valve	Z95.2*
Xeno Heart Valve	Z95.3*
Pacemaker AICD/VAD	Z95.0*, Z95.81*
Pulmonary Hypertension	I27.2*, I27.0*, I27.81, I27.83
Any CKD	N18*
Stage 1 CKD	N18.1*
Stage 2 CKD	N18.2*
Stage 3 CKD	N18.3*
Stage 4 CKD	N18.4*
Stage 5 CKD	N18.5*
ESRD	N18.6*, Z99.2*, T85.631*
Kidney Transplant	Z94.0*
Liver Transplant	Z94.4*
Lung Transplant	Z94.2*
Heart Transplant	Z94.1*
Pancreas Transplant	Z94.83*
Afib flutter	I48*
VTach VFib	I47.0*, I47.2*, I49.0*
Supraventricular Tachycardia	I47.1*
Prior Cardiac Arrest	I46*
Cerebrovascular Disease	G45.*, G46.*, H34.0, I6*.*, I97.81*, I97.82*
Osteoporosis	M81*
Ethanol Abuse	F10*, K70*, G62.1*, I42.7*, K29.2*, O35.4*, O99.31
HIV	B20*, B97.35*, R75*, Z21*
Avian Swine Flu	J09*
IBD	K50*, K51*
Sleep Apnea	G47.3*
Lupus	L93*, M32*
Rheumatoid Arthritis	M06*, M05*
Any Cancer	C**.*, C44.*
Breast Cancer	C50*
Lung Cancer	C34.1*, C34.2*, C34.3*, C34.8*, C34.9*

Metabolic Syndrome	E88.81*
Previous Bariatric Surgery	Z98.84*

Abbreviations: T1DM: Type 1 diabetes mellitus, T2DM Type 2 diabetes mellitus, HFpEF: heart failure with preserved ejection fraction, HF: heart failure, CAD: coronary artery disease, NAFLD: non-alcoholic fatty liver disease, NASH: non-alcoholic steatohepatitis, VTE: venous thromboembolism, HIT: heparin induced thrombocytopenia, DIC: disseminated intravascular coagulation, ITP: idiopathic thrombocytopenia, HLH: hemophagocytic lymphohistiocytosis, MI: myocardial infarction, COPD: chronic obstructive lung disease, ILD: interstitial lung disease, AICD: Automatic Implantable Cardioverter Defibrillator, VAD: ventricular assist device, CKD: chronic kidney disease, ESRD: end stage renal disease, Afib: atrial fibrillation, HIV: human immunodeficiency virus, Flu: influenza, IBD: inflammatory bowel disease.
