# Supplementary material for: Characterizing COVID-19 clinical phenotypes and associated comorbidities and complication profiles
Source: PLoS One. 2021 Mar 31;16(3):e0248956. doi: 10.1371/journal.pone.0248956 (PMC8011766; doi:10.1371/journal.pone.0248956)
Supplement: S2 Table — (PDF) [file pone.0248956.s011.pdf]

S2 Table: List of Complications contributing to each Complication category.

Category	Complication
Cardiovascular	Hypotension (sustained SBP < 90)
	Atrial fibrillation
	Acute Heart Failure
	Myocardial infarction
	BNP (BNP > 450 after initial 48 hours of admission)
	Troponemia (Troponin > 0.5 after initial 48 hours of admission)
Respiratory	Pneumothorax
	Pulmonary Edema
	Acute respiratory distress syndrome
	Respiratory Failure
	Healthcare associated pneumonia
	Ventilator associated pneumonia
Hematologic	In-hospital bleeding event
	Cerebrovascular accident
	Myocardial infarction
	Pulmonary embolism
	Deep venous thrombosis
	Severe Anemia (Hgb < 6)
Renal	Urinary tract infection
	Acute Renal Failure
	1.0 point increase in creatinine from baseline after initial 48 hours in hospital
Metabolic	Rhabdomyolysis
	Malnutrition
	Hypernatremia (>149)
	Hyperkalemia (>5)
	Hypoglycemia (< 60)
	Hyperglycemia (> 180)
Hepatic	AST > 200 after initial 48 hours
	Total bilirubin > 3 after initial 48 hours
Infectious	Bacteremia
	Clostridium difficile infection
	Sepsis
	Urinary tract infection
	Healthcare associated pneumonia
	Ventilator associated pneumonia

Abbreviations: SBP: systolic blood pressure, BNP: beta natriuretic peptide, Hgb: hemoglobin, AST: aspartate transaminase.
