# Supplementary material for: Characterizing COVID-19 clinical phenotypes and associated comorbidities and complication profiles
Source: PLoS One. 2021 Mar 31;16(3):e0248956. doi: 10.1371/journal.pone.0248956 (PMC8011766; doi:10.1371/journal.pone.0248956)
Supplement: S3 Table — (PDF) [file pone.0248956.s012.pdf]

S3 Table 3: Association of Clinical Phenotype with In-Hospital Complications

Complications*	OR	95% CI	P value
**Cardiovascular**			0.93 (LR test)
Phenotype I	0.90	0.42 – 1.91	0.78
Phenotype II	1.00	0.53 – 1.89	1.00
**Respiratory**			<0.001 (LR test)
Phenotype I	2.98	1.58 – 5.59	<0.001
Phenotype II	2.32	1.29 – 4.17	0.01
**Hematologic**			0.02 (LR test)
Phenotype I	2.11	0.99 – 4.48	0.05
Phenotype II	0.99	0.48 – 2.04	0.97
**Renal**			<0.001 (LR test)
Phenotype I	7.04	3.11 – 15.9	<0.001
Phenotype II	2.57	1.15 – 5.74	0.02
**Hepatic**			<0.001 (LR test)
Phenotype I	8.35	1.93 – 36.11	<0.001
Phenotype II	0.56	0.1 – 3.09	0.51
**Metabolic**			<0.001 (LR test)
Phenotype I	4.85	2.78 – 8.45	<0.001
Phenotype II	2.57	1.52 – 4.34	<0.001
**Infectious**			<0.001 (LR test)
Phenotype I	2.57	1.57 – 4.21	<0.001
Phenotype II	1.51	0.96 – 2.38	0.07

Abbreviations: OR, odds ratio; CI, confidence interval; LR, likelihood ratio

** Reference group for all models is Phenotype III. All models adjusted for sex, race/ethnicity, and Elixhauser Comorbidity Index.*
