# Supplementary material for: Characterizing COVID-19 clinical phenotypes and associated comorbidities and complication profiles
Source: PLoS One. 2021 Mar 31;16(3):e0248956. doi: 10.1371/journal.pone.0248956 (PMC8011766; doi:10.1371/journal.pone.0248956)
Supplement: S4 Table — (PDF) [file pone.0248956.s013.pdf]

	LOS IRR with in-hospital death included	LOS IRR with in-hospital death excluded
**Cluster 1 (vs ref Cluster 3)**	1.68 (95% CI 1.41-2.01)	1.74 (95% CI 1.45-2.1)
**Cluster 2 (vs ref Cluster 3)**	1.23 (95% CI 1.07-1.44)	1.22 (95% CI 1.05-1.43)

IRR = incidence rate ratio; LOS = length of stay

Sensitivity analysis to evaluate the impact of in-hospital mortality on hospital length of stay (LOS). Negative binomial regression was used in this analysis. No difference was noted in length of stay with or without accounting for in-hospital mortality.
