# Supplementary material for: Characterizing COVID-19 clinical phenotypes and associated comorbidities and complication profiles
Source: PLoS One. 2021 Mar 31;16(3):e0248956. doi: 10.1371/journal.pone.0248956 (PMC8011766; doi:10.1371/journal.pone.0248956)
Supplement: S5 Table — (PDF) [file pone.0248956.s014.pdf]

S5 Table: Home medications and hospital day 5 laboratory values of hospitalized COVID-19 patients with clinical phenotypes I, II, and III.

	**Phenotype I**	**Phenotype II**	**Phenotype III**	**P value**
	N=236	N=613	N=173	
**Home Medications***				
Warfarin	29.5 (8.9)	30.8 (8.2)	30.4 (13.4)	0.21
Montelukast	9 (3.8%)	47 (7.7%)	17 (9.8%)	0.05
Inhaled Ipratropium	2 (0.8%)	29 (4.7%)	6 (3.5%)	0.03
Digoxin	13 (5.5%)	43 (7.0%)	15 (8.7%)	0.46
Oral Steroids	2 (0.8%)	6 (1.0%)	3 (1.7%)	0.65
Proton Pump Inhibitor	30 (12.7%)	108 (17.6%)	37 (21.4%)	0.06
Clopidogrel	57 (24.2%)	181 (29.5%)	44 (25.4%)	0.23
Inhaled Steroids	9 (3.8%)	19 (3.1%)	8 (4.6%)	0.61
Nasal Fluticasone	17 (7.2%)	79 (12.9%)	26 (15.0%)	0.03
Aspirin	19 (8.1%)	87 (14.2%)	34 (19.7%)	0.003
Amiodarone	3 (1.3%)	6 (1.0%)	4 (2.3%)	0.38
Albuterol	71 (30.1%)	182 (29.7%)	55 (31.8%)	0.87
Loop Diuretic†	1 (0.4%)	5 (0.8%)	4 (2.3%)	0.13
Antihistamine	34 (14.4%)	146 (23.8%)	46 (26.6%)	0.004
Statin	38 (16.1%)	106 (17.3%)	25 (14.5%)	0.66
Metformin	12 (5.1%)	71 (11.6%)	22 (12.7%)	0.01
ACE inhibitor	81 (34.3%)	221 (36.1%)	60 (34.7%)	0.87
ARB	16 (6.8%)	50 (8.2%)	16 (9.2%)	0.65
**Hospital Day 5 Labs***				
**Hematologic**				
PLT	264.0 (188.0-342.0)	255.5 (184.0-342.0)	210.0 (159.0-264.0)	0.002
PTT	40.0 (32.0-65.0)	33.5 (30.0-42.5)	32.5 (31.0-40.5)	0.02
Fibrinogen	503.5 (422.0-648.0)	565.0 (470.0-684.0)	476.0 (418.0-573.0)	0.01
D-Dimer	3.1 (1.9-6.1)	1.1 (0.6-1.9)	0.8 (0.4-1.3)	<0.001
Hematocrit	33.1 (28.5-36.7)	36.4 (32.9-40.3)	36.3 (32.2-40.7)	<0.001
**Inflammatory**				
CRP	76.5 (29.0-173.0)	54.0 (19.0-134.5)	20.0 (5.0-46.0)	<0.001
ESR	29.0 (28.0-48.0)	76.5 (41.0-113.0)	52.0 (2.0-102.0)	0.31
IL-6	190.5 (62.4-749.0)	62.6 (20.7-472.0)	31.1 (6.9-264.1)	0.09
IL-8	54.3 (39.6-163.5)	56.1 (36.3-112.0)	24.3 (8.1-40.4)	0.18
TNF alpha	26.8 (21.5-36.7)	33.5 (17.8-40.6)	16.7 (7.2-26.2)	0.36
IL-1B	0.8 (0.4-1.4)	0.8 (0.3-1.3)	0.3 (0.0-0.7)	0.40
LDH	390.5 (318.0-519.0)	338.0 (263.0-436.0)	263.0 (182.0-331.0)	<0.001
Ferritin	774.0 (490.0-2165.0)	637.5 (256.0-1757.5)	330.0 (162.0-870.5)	0.01
**Cardiovascular**				
EKG QTc	382.0 (342.0-420.0)	371.0 (348.0-404.0)	349.0 (304.0-418.0)	0.52
Pro-BNP	89.0 (25.0-434.0)	167.0 (52.0-365.0)	507.0 (55.5-1083.5)	0.62
Lactate	1.3 (1.0-1.8)	1.2 (0.9-1.7)	1.6 (0.9-2.1)	0.41
**Other**				
AST	44.0 (28.0-75.0)	40.0 (28.0-59.0)	27.0 (17.0-41.0)	0.002
Bicarbonate	26.0 (23.0-28.0)	25.0 (23.0-27.0)	26.0 (24.0-29.5)	0.01

Procalcitonin	0.6 (0.2-1.4)	0.3 (0.1-0.7)	0.1 (0.1-0.2)	<0.001
Albumin	2.0 (1.7-2.5)	2.5 (2.1-2.8)	3.0 (2.5-3.4)	<0.001
Total Bilirubin	0.6 (0.4-0.9)	0.5 (0.3-0.7)	0.5 (0.3-0.6)	0.005
Triglyceride	210.0 (151.0-297.0)	184.0 (128.0-341.0)	53.0 (17.0-79.0)	0.02
Glucose	134.0 (107.0-176.0)	122.0 (98.0-157.0)	106.5 (94.0-132.0)	<0.001
Creatinine	0.9 (0.7-1.6)	0.8 (0.7-1.2)	0.8 (0.7-1.0)	0.03
WBC	9.2 (7.3-12.1)	7.1 (5.3-9.4)	5.8 (4.7-7.4)	<0.001
ANC	8.1 (5.6-10.4)	5.6 (3.8-7.7)	3.9 (2.1-5.6)	<0.001
ALC	1.0 (0.6-1.5)	1.0 (0.7-1.6)	1.3 (0.9-1.8)	0.019

* Categorical variables presented as count (%), continuous variables presented as median (interquartile range).

† Loop Diuretics include furosemide, torsemide, budesonide.

Abbreviations: ALC, Absolute Lymphocyte Count; ANC, Absolute Neutrophil Count; ARB, Angiotensin receptor blocker; AST, Aspartate transaminase; CRP, C-Reactive Protein; ESR, Erythrocyte Sedimentation Rate; EKG, Electrocardiogram; IL, Interleukin; LDH, Lactate Dehydrogenase; Pro-BNP, proB-type Natriuretic Peptide; PLT, Platelets; PTT, Partial Thromboplastin Time; TNF-alpha, Tumor Necrosis Factor-alpha; WBC, White Blood Cell;
