# Supplementary material for: Characterizing COVID-19 clinical phenotypes and associated comorbidities and complication profiles
Source: PLoS One. 2021 Mar 31;16(3):e0248956. doi: 10.1371/journal.pone.0248956 (PMC8011766; doi:10.1371/journal.pone.0248956)
Supplement: Attachment — Submitted filename: PONE.docx [file pone.0248956.s016.docx]

1. The study presents the results of original research.

It is OK.
2. Results reported have not been published elsewhere.

NOT
3. Experiments, statistics, and other analyses are performed to a high technical standard and are described in sufficient detail.

YES
4. Conclusions are presented in an appropriate fashion and are supported by the data.

More transparency is needed for this part.

5. The article is presented in an intelligible fashion and is written in standard English.

Mention the study interval.

6. The research meets all applicable standards for the ethics of experimentation and research integrity.

If it is according to the Journal format, there is no problem.
7. The article adheres to appropriate reporting guidelines and community standards for data availability.

Complete the description of the tables.
